# Promiscuous stimulation of HSP70 ATPase activity by parasite‐derived J‐domains

**DOI:** 10.1002/2211-5463.70207

**Published:** 2026-01-31

**Authors:** Julian Barth, Moritz Koch, Le‐Han Rössner, Johanna Eichhorn, Denys Pogoryelov, Matthias P. Mayer, Jude M. Przyborski

**Affiliations:** ^1^ Biochemistry and Molecular Biology Justus Liebig University Giessen Germany; ^2^ Center for Molecular Biology (ZMBH) Heidelberg Germany; ^3^ Institute of Botany Justus Liebig University Giessen Germany; ^4^ Institute of Pharmaceutical Chemistry Goethe‐University Frankfurt Frankfurt am Main Germany

**Keywords:** ATPase activity, HSP70, J‐domain protein, molecular chaperone, parasite–host interaction, *Plasmodium falciparum*

## Abstract

The malaria parasite *P. falciparum* exports a large number of proteins to the human host cell, including members of the J‐domain protein (JDP) family. In other systems, JDPs stimulate the ATPase activity of HSP70 and direct client protein interactions. Three exported *Pf*JDP are highly homologous yet appear to have divergent roles. We examined the ability of isolated J‐domains from these proteins to stimulate the ATPase activity of *Pf*HSP70‐X and human *Hs*HSP70 and *Hs*HSC70. *In vitro* assays demonstrate that all domains stimulate the ATPase activity of each HSP70 tested. While overall stimulation was broadly comparable, differences were observed between individual *Pf*JDP–HSP70 pairings. Our findings support a model in which *the parasite systematically* exports JDPs to exploit the host's chaperone power.

## Graphical abstract

The malaria parasite *Plasmodium falciparum* exports three highly homologous yet functionally divergent J‐domain proteins into human erythrocytes. Here, we show that J‐domains isolated from all three proteins effectively stimulate the ATPase activity of both endogenous host and exported parasite HSP70 chaperones. These findings support a hypothesis in which the parasite hijacks the human chaperone machinery for its own benefit.

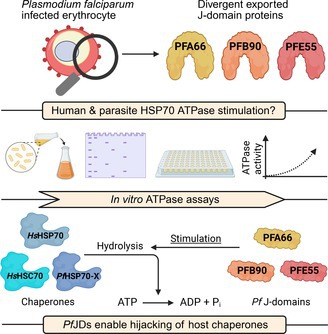

**Abbreviations**
aiH: autoinhibitory helix
BioID: biotin identification
CV: column volume
DD: dimerisation domain
FI: fluorescence intensity
G/F‐region: glycin/phenylalanine rich region
HSF1: heat shock transcription factor 1
HSP70: heat shock protein 70
IPTG: isopropyl‐β‐D‐1‐thiogalactopyranoside
ipTM: interface predicted template modellingmodeling score
JDP: J‐domain protein
NEF: nucleotide exchange factor
PEXEL: *Plasmodium* export element
pTM: predicted template modellingmodeling score
RBCC: red blood cell cytosol
RT: room temperature
SBD: substrate binding domain
ULP1: ubiquitin‐like specific protease 1
βSD: β‐sandwich domains

The parasite *Plasmodium falciparum* causes the most severe form of malaria in humans, leading to over 500 000 deaths per year [1]. The severity of *P. falciparum* infection compared to other forms of malaria is due in large part to the parasite's ability to modify its host cell, the mature human erythrocyte. This leads to cytoadherence, in which infected erythrocytes stick to endothelial cells lining blood vessels, including those within organs and the brain [2]. Over the past decades, the mechanisms underlying this phenomenon have been studied and have revealed that proteins exported from the parasite to the host cell directly or indirectly support host cell modification [3, 4]. The parasite exports approximately 10% of its entire proteome, highlighting the importance of this process [5, 6, 7]. Of particular note, amongst the exportome are 18 proteins belonging to the J‐domain protein (JDP) family [8, 9]. Such proteins have been characterised in detail in other systems and fulfil their function in concert with chaperones belonging to the Heat Shock Protein 70 (HSP70) family. JDPs are important in recruiting substrate proteins to HSP70 and additionally act to stimulate the ATPase activity of HSP70, leading to diverse downstream effects [10, 11, 12, 13]. Stimulation of HSP70 by JDP is dependent on an HPD motif within the J‐domain, and this motif is essential for the concerted action of JDP with HSP70 [14]. Of the 18 JDP proteins predicted to be exported by the malaria parasite to the host cell (referred to here as *Pf*JDP), the majority contain a corrupted HPD motif, have thus been classified as Type IV JDP [8], and their molecular functions remain elusive. Of the HPD‐containing exported *Pf*JDP, one small subfamily containing three proteins has been studied in detail. These Type II JDP, encoded by PF3D7_0113700, PF3D7_0501100, PF3D7_0201800 (also referred to as respectively PFA66, PFE55 and PFB90/KaHSP40), share high sequence homology, particularly in their J‐domain (Fig. 1A,B and Data S1); however, they have differential functions within the host cell. PFA66 and PFE55 have been localised to J‐dots, highly mobile structures within the host cell, while PFB90 appears to localise to the knobs, electron‐dense structures at the plasma membrane of the infected cell, playing a key role in cytoadherence (Fig. 1A) [15, 16, 17, 18]. Knockouts of each gene also show divergent results. While a knockout of *pfa66* leads to aberrant knob structures and a loss of cytoadherence, knockouts of either *pfb90* or *pfe55* show no detectable mutant phenotype, suggesting a differential function for each member of the subfamily [4, 19]. The difference in result between knockouts of *pfa66* and *pfe55* is particularly striking as the gene products appear to have an identical localisation [15]. The host erythrocyte contains, in addition to a parasite‐encoded exported HSP70 (*Pf*HSP70‐X), substantial amounts of residual human HSP70s [16, 20] (Fig. 1A). Interestingly, a knockout of *pfhsp70x* leads to only minimal effects on host cell modification, while the *pfa66* knockout shows severe effects [19, 21, 22]. Taken together, these data suggests that PFA66 exerts its important function in concert with a residual human HSP70, and that this interaction cannot be complemented by PFB90 or PFE55, implying specificity in the *Pf*JDP‐HSP70 interaction. We and others have previously demonstrated both physical and functional interactions between exported *Pf*JDP and these molecular chaperones [18, 19, 23, 24, 25]. However, these studies used variant assay conditions and utilised different domain combinations of the *Pf*JDP, leading to divergent results. Additionally, these previous studies were not able, or designed to, differentiate between a true JDP‐HSP70 interaction (via the HPD motif), recognition of *Pf*JDPs (or domains hereof) as HSP70 substrates, or differing affinity between the J‐domains and their HSP70 partner, further muddying the result water. To finally clarify the situation, in this study, we use a well‐established *in vitro* assay format that allows us to distinguish between these two modes of HSP70 activation and exclude that any detected HSP70 ATPase activity is due to a nonphysiological substrate/chaperone interaction [26, 27]. We assayed the stimulatory effect of the three J‐domains on three HSP70s under identical conditions. Our data reveal that J‐domains from PFA66, PFE55 and PFB90 are all able to effectively stimulate the ATPase activity of *Pf*HSP70‐X and two residual human HSP70s. Our data also reveal small differences between the ability of the J‐domains to stimulate each individual HSP70, but a significant difference between their stimulatory effects on specific HSP70s. These data have implications for a general understanding of the functional J‐domain/HSP70 interaction. Furthermore, our data offer a deeper understanding of this unusual molecular host–parasite interaction and offers further insight into the specific functions of exported *Pf*JDP.

**Fig. 1:**
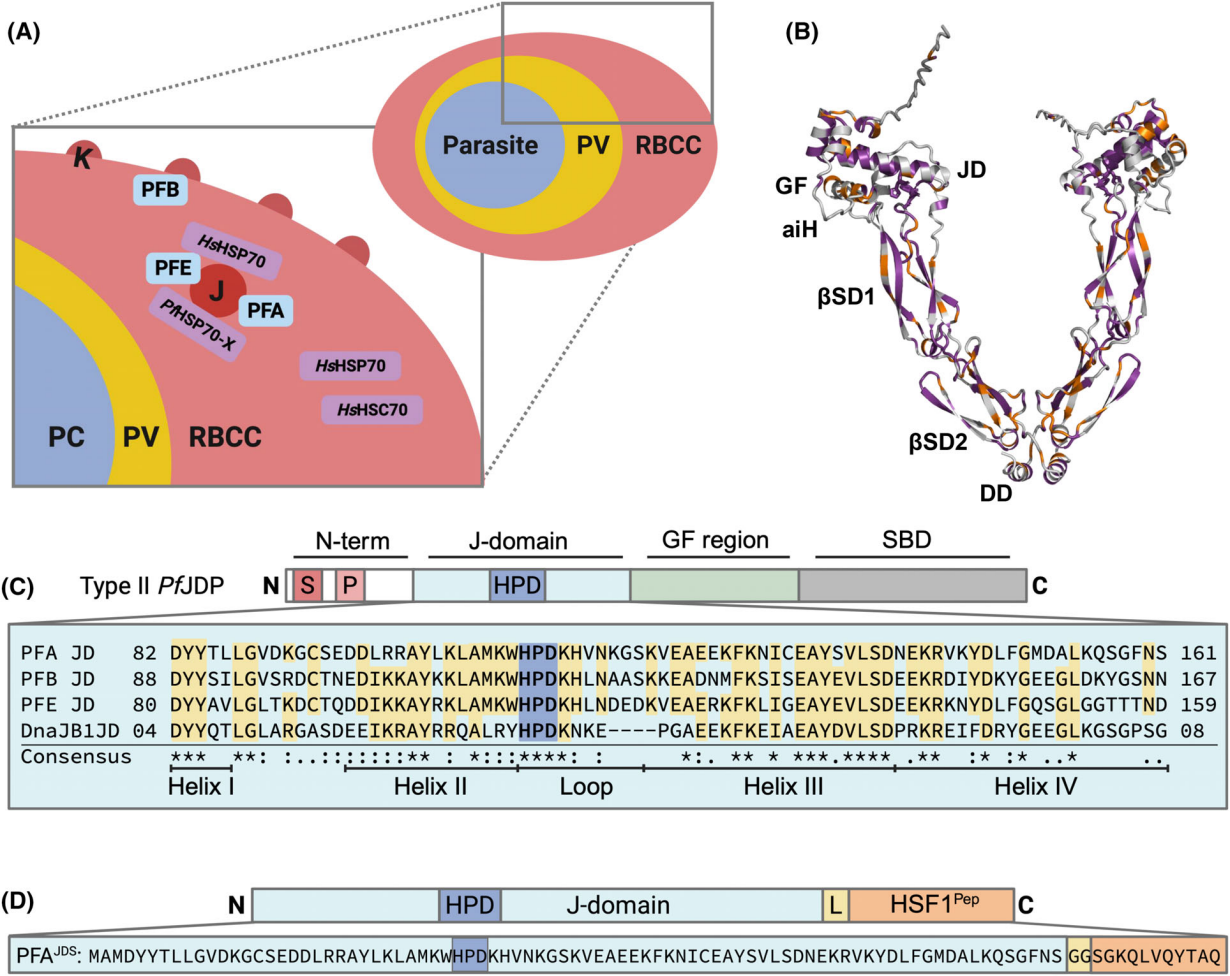
(A) Known subcellular localisations of chaperones and co‐chaperones used in this study. The malaria parasite resides within mature human erythrocytes, separated from the erythrocyte cytosol by a membrane‐bound parasitophorous vacuole (PV). The parasite modifies the host cell, including the biogenesis of knobs below the surface of the erythrocyte plasma membrane (K), and J‐dots in the erythrocyte cytosol (J). The erythrocyte contains the residual human chaperones *Hs*HSP70 and *Hs*HSC70 and the parasite‐encoded *Pf*HSP70‐X. *Pf*HSP70‐X has been localised to, and *Hs*HSP70 associated with, the J‐dots. The exported *Pf*JDPs PFA66 (PFA) and PFE55 (PFE) have been localised to the J‐dots, and PFB90 (PFB) to the knobs. PC, parasite cytosol; PV, parasitophorous vacuole; RBCC, red blood cell cytosol. Figure generated with BioRender. (B) Alpha‐Fold secondary structure representation of the predicted structures of PFA66 coloured according to the conservation between the three JDPs with violet‐purple complete sequence identity and orange conservative replacements as defined by ([H,K,R], [D,E,N,Q], [S,T], [I,L,V,F], [F,Y,W]). Predicted domains are labelled as: JD, J‐domain; GF, glycine‐phenylalanine‐rich region; aiH, autoinhibitory helix; βSD1 and βSD2, β‐sandwich domains; DD, dimerisation domain. (C) Upper: General domain structure of the exported *Pf*JDP. The N‐term domain contains an ER‐type hydrophobic signal sequence (S), followed by a PEXEL export sequence (P) which directs the protein to the host cell. Following this is a J‐domain, containing the HPD motif essential for stimulation of HSP70, a glycine/phenylalanine‐rich region (GF), and a C‐terminal substrate binding domain (SBD). Lower: alignment of J‐domains of the proteins of interest (POI). As a comparison, we include the J‐domain from *Hs*DnaJB1, a known co‐chaperone for human HSP70s. Asterisks indicate identical amino acids. (D) Structure of the fusion proteins used in this study (using PFA^JDS^ as an example). The J‐domain from the POI is fused N‐terminal to a short flexible linker (L) and the peptide sequence derived from HSF1 (HSF1^pep^) that binds with high affinity to *Hs*HSC70 [27]. Figures A, C, D were generated using BioRender.

## Materials and methods

### Nomenclature

We refer to the HSP70s used in our assays using the following nomenclature: *Pf*HSP70‐X (PF3D7_0831700); *Hs*HSP70 (HSPA1, NP_005336.3); *Hs*HSC70 (HSPA8, NP_006588.1).

### Plasmids

Full‐length codon‐optimised *P. falciparum* JDPs sequences (PFA: PF3D7_0113700, PFB: PF3D7_0201700, PFE: PF3D7_0501100) were ordered from GeneScript (Piscataway, NJ, USA). Sequences for PFA^JDS^, PFB^JDS^, PFE^JDS^ and QPD mutants were generated by overlap extension PCR (Primers in Data S9) and cloned into the pSUMO vector using MscI and SalI (New England Biolabs, Frankfurt a. M., Germany). pSUMO plasmids containing coding sequences for *Hs*HSP70, *Hs*HSC70 and *Pf*HSP70‐X have been previously described [19].

### Heterologous overexpression

Plasmids were transformed into BL21 pRARE II *E. coli*. Proteins of Interest (POI) were expressed as N‐terminal SUMO‐His^6^‐tagged proteins. Single colonies were used to inoculate 3 mL of LB medium and incubated at 37 °C for 4–6 h including 50 μg·mL^−1^ kanamycin and 17 μg·mL^−1^ chloramphenicol. This pre‐culture was used to inoculate 150 mL of 2xYT medium and incubated overnight at 37 °C including appropriate antibiotics. 40 mL of this overnight culture was used to inoculate 1 L of 2xYT medium and grown at 37 °C. At OD_600_ 0.8 protein expression was initiated using 1 mm IPTG and the culture was grown for 16 h at RT. Cells were harvested via centrifugation at 20 000 ***g*** for 15 min at 4 °C. The cell pellet of 1 L culture was resolubilised in 40 mL chilled lysis buffer (J‐domain: 50 mm Tris/HCl, 500 mm NaCl, 5 mm MgCl_2_, pH 7.9; HSP70: 50 mm Tris/HCl, 100 mm KCl, 5 mm MgCl_2_, pH 7.9) supplemented with protease inhibitors (Pepstatin 600 nm, E64 5 μm, PMSF 1 mm) and stored at −20 °C.

### Purification of recombinant POI

Purification was carried out according to literature with slight modifications [19]. For lysis, cell pellets were subjected to one freeze–thaw cycle, supplemented afterwards with lysozyme and DNase I and stirred for 1 h at 4 °C. This temperature was used for all following purification steps if not indicated otherwise. Lysis was followed by mild sonication and the supernatant clarified by centrifugation (20 000 ***g***, 30 min).

The supernatant was mixed with 2 g of Ni‐IDA beads (Macherey‐Nagel, Düren, Germany) and incubated for at least 1 h. The supernatant and the column material were transferred into a glass column (J‐domain: 15 mL volume; HSP70: 70 mL volume) separated by gravity flow and subsequently washed with 10 column volumes (CV) of the respective lysis buffer. For the second washing step, to remove potential DnaK contamination, 10 CV of lysis buffer were supplemented with 5 mm ATP. The first half of this buffer was allowed to run through, then the column was closed, and the mixture incubated for 30 min. Afterwards, the remaining ATP buffer was allowed to run through. To further flush ATP from the system, another washing step with 10 CV lysis buffer was performed. The proteins of interest were slowly eluted using 10 mL lysis buffer supplemented with 500 mm imidazole. The eluted fraction was mixed with 8 mg ULP1 protease (produced in the Przyborski laboratory, expression vector was a kind gift of Matthias Mayer). Dialysis followed against 2 L dialysis buffer [J‐domain: 40 mm HEPES pH 7.6, 300 mm KCl, 5 mm MgCl_2_, 2 mm DTT, 10% glycerin, 8 kDa MW cut‐off tube (Spectrum Laboratories, Rancho Dominguez, CA, USA); HSP70: 40 mm HEPES pH 7.6, 150 mm KCl, 5 mm MgCl_2_, 10% glycerin, 50 kDa MW cut‐off tube (Repligen, Waltham, MA, USA)], supplemented with 2 mm DTT overnight. The SUMO‐His^6^‐tag is proteolytically cleaved from the proteins of interest. The next day, the protein solution was incubated with 2.5 g of fresh Ni‐IDA beads for 1 h, the mixture filled into a glass column, and POI were separated from ULP1 and SUMO‐His‐Tag by allowing the solution to run through by gravity flow. Then, 10 mL of dialysis buffer were used to flush out residual POI. Fractions containing the POI (confirmed by SDS/PAGE, digitised via scanning) were either pooled, aliquoted and stored at −80 °C, or prepared for size exclusion chromatography.

Size exclusion chromatography was carried out for all HSP70s and J‐domain fusion proteins to improve their purity and remove residual nucleotides. POI were centrifuged (10 000 ***g***, 15 min) to remove aggregates from the solution, then loaded on a HiLoad™ 16/600 Superdex™ 200 pg column and purified with an ÄKTA pure™ FPLC system coupled to an F9‐R fraction collector tube rack and monitored by unicorn software version 7.2 (Cytiva, Uppsala, Sweden). Fractions containing only the POI (confirmed by SDS/PAGE) were pooled. Protein concentration was measured using the Bradford method. Aliquots were stored at −80 °C.

### ATPase assay (steady state)

Steady‐state ATPase measurements were performed using the Transcreener® ADP^2^ fluorescence intensity (FI) assay (Bellbrooks Labs, Madison, WI, USA) according to the manufacturer's instructions with slight modifications. To optimise HSP70 concentration and check the linearity and upper limits of the assay, an enzyme titration in ATPase buffer was performed (Data S4B). To assess ATPase activity, the procedure was split into two parts, both carried out in black 384‐well plates (Greiner Bio‐One, Kremsmünster, Austria, REF#784076). The first step was the enzymatic ATPase reaction in ATPase buffer (40 mm Hepes pH 7.6, 5 mm MgCl_2_, 100 mm KCl). Aliquots of *Hs*HSP70, *Hs*HSC70 and *Pf*HSP70‐x were thawed on ice. The enzymatic reaction was prepared by mixing 1 μm HSP70 with either no cofactors, 1 μm HSF1 peptide, 1 μm of the PFA^JDS^, PFB^JDS^ or PFE^JDS^ fusion proteins or their corrupted versions PFA^JDSQ^, PFB^JDSQ^ or PFE^JDSQ^. The enzymatic reaction was initiated by adding 100 μm ATP and the mixture was incubated at 37 °C for 1 h. In the second step, the enzymatic reaction was terminated by an equal amount of freshly prepared detection mixture (40 mm EDTA, 20 mm HEPES, 0.02% Brij‐35, pH 7.5, 8 nm ADP Alexa Fluor® 594 Tracer, 93.7 μg·mL^−1^ ADP^2^ Antibody‐IRDye® QC‐1). The reaction mixture was incubated in the Tecan Infinite M200 Pro at RT for 1 h and i‐control software (version 3.9.10) was programmed to automatically follow up with fluorescence signal development and the readout. The excitation wavelength was 580 nm and the emitted signal was measured at a wavelength of 625 nm.

### ATPase assay (single turnover)

Single turnover ATPase assays were performed on *Hs*HSP70, *Hs*HSC70 and *Pf*HSP70‐X as previously described [11, 19] with slight modifications. First, 50 μL of 50 μm HSP70 in reaction buffer (Tris/HCl 50 mm, pH 7.6, KCl 150 mm, MgCl_2_ 10 mm, DTT 2 mm) were mixed with 2 μL of ATP mixture (20 mm ATP + 1 μL [α^32^P] ATP [10 μCi·μL^−1^]). The resulting mixture was allowed to form complexes on ice for 2 min, then HSP70‐ATP complexes were separated from unbound nucleotides by gel filtration on a NICK column at RT. Fractions were collected drop‐wise on ice. The first four fractions containing radioactivity (detected by Geiger counter) were pooled, aliquoted, snap frozen and stored at −80 °C.

For activity measurements, HSP70‐ATP complexes were thawed and 0.5 μL withdrawn for the *t* = 0 time point. The reaction was started by diluting 6 μL of the complexes in 44 μL of reaction buffer. The buffer was supplemented with either no cofactors, 1 μm HSF1 peptide, or 1 μm of the J‐domain fusions. Over 20 min, 2 μL samples were withdrawn and spotted on a thin layer chromatography plate (PEI cellulose, Merck, Darmstadt, Germany), which was prespotted with 5 μL of 5 mm ATP/ADP mixture. The plates were developed in 400 mm LiCl in 10% acetic acid to separate the nucleotides and exposed to fluoroimaging screens overnight. A Fuji FLA phosphoimager was used to quantify relative ATP and ADP amounts.

### Data evaluation

graphpad prism version 10.5.0 was used for calculating statistics. The test performed was an ordinary one‐way ANOVA with Holm–Šídák's multiple comparisons modification. Statistical significance was defined as follows: *P* > 0.05 = nonsignificant (ns); *P* < 0.05 = *; *P* < 0.01 = **; *P* < 0.001 = ***.

### Molecular modelling

Protein–protein complexes [HSP70 and J‐domain variants (PFA^JDS^, PFB^JDS^, PFE^JDS^)] were modelled using AlphaFold 3 (DeepMind/Isomorphic Labs) via the AlphaFold web server (https://alphafoldserver.com). Predictions were performed in multimer mode with default 10 recycles. Model quality was evaluated by pTM (predicted TM‐score), ipTM (interface predicted TM‐score), and an overall ranking score combining these metrics. Additional quality descriptors included fraction of predicted disordered residues, presence of steric clashes and per‐chain interaction confidence.

## Results

### Modelling of PFA66, PFB90 and PFE55

To better understand the difference between the three class II *Pf*JDPs, we used AlphaFold 3 to model the tridimensional structure of the three *Pf*JDPs (Fig. 1B, Data S2). For all three *Pf*JDPs, the confidence of the structure prediction was highest in the C‐terminal region (PFA66 165–342; PFB90 175–354; PFE55 163–340; amino acid numbers relative to the PEXEL cleavage site), which contains the two homologous β‐sandwich domains and the C‐terminal dimerisation domain, which are typical for class B JDPs. The confidence of prediction was slightly lower in the J‐domain but very low in the glycine‐phenylalanine‐rich linker region (G/F‐region), which joins the J‐domain to the β‐sandwich domain 1 and contains the autoinhibitory helix [28]. The G/F‐rich region is of different length in the three JDPs (PFA66 76; PFB90 88; PFE55 78), which is likely the reason for the different position of the J‐domain relative to the remainder of the protein in the prediction, and this position may be rather arbitrary. Therefore, as expected from the high degree of sequence identity (Fig. 1C and Data S1), the structure of the three JDPs is highly similar and does not offer an obvious explanation for the difference in the physiological role they play in *Plasmodium falciparum*‐infected red blood cells.

### Design and production of recombinant proteins for the ATPase assay

ATPase activity of HSP70 can be stimulated both by binding of a substrate and by interaction with a J‐domain to a lower degree. In the simultaneous presence of both a J‐domain and a protein substrate, the ATPase activity of HSP70s is synergistically stimulated up to 100‐fold [10, 29, 30, 31]. In full‐length JDPs, sequences downstream of the J‐domain may act as a substrate and thus some JDPs could deliver both signals: from the J‐domain and the substrate [10]. This confounds the measurements of the ATPase stimulation by full‐length JDPs. Any differences observed may be due to sequences outside the J‐domain and may be nonphysiological when the JDP has a substrate bound. To avoid this complication and to create comparable situations for all JDPs, we generated constructs to express isolated J‐domains derived from *Pf*JDPs, fused to a peptide sequence derived from the human heat shock transcription factor HSF1, known to be recognised by human HSP70 as a substrate [27], to simulate a JDP‐substrate complex able to synergistically stimulate the ATPase activity of HSP70s [19] (Fig. 1C). As a control, we generated constructs containing a mutated HPD motif (QPD), which lacks the J‐domain signal and should not be able to synergistically stimulate the ATPase activity. Recombinant *Pf*HSP70‐X, *Hs*HSP70 (HSPA1) and *Hs*HSC70 (HSPA8) were produced as previously described [19]. Recombinant proteins produced in *E. coli* were purified to homogeneity (Data S3) and verified to be free of contaminating DnaK (data not shown).

### HSP70 ATPase stimulation by recombinant J‐domains under steady state conditions

**Fig. 2:**
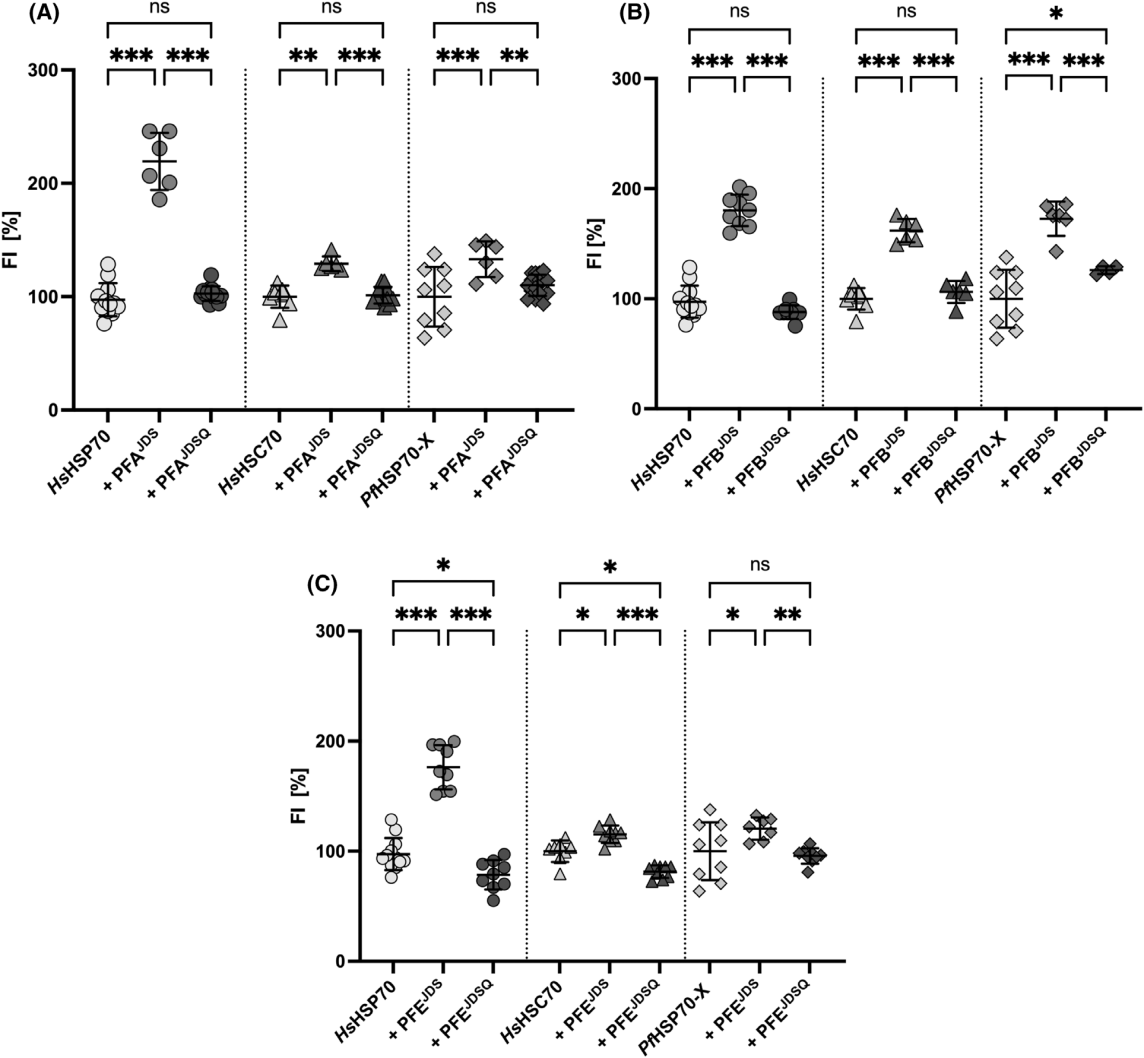
Stimulation of HSP70 ATPase activity by *Pf*JD (steady state conditions). All values were normalised against the basal ATPase rate of the HSP70 alone (set to 100%). For all assays *n* ≥ 3, with error bars indicating SD. Statistics were performed with ordinary one‐way ANOVA with Holm‐Šídák's multiple comparisons modification. *P* > 0.05 = nonsignificant (ns); *P* < 0.05 = *; *P* < 0.01 = **; *P* < 0.001 = ***. All assays included a control using a nonfunctional mutated version of the J‐domain containing a QPD motif (JDSQ). (A) Stimulation of HSP70s by PFA^JDS^. (B) Stimulation of HSP70s by PFB^JDS^. (C) Stimulation of HSP70s by PFE^JDS^.

We tested the ability of the recombinant J‐domain fusions to stimulate the ATPase activity of *Pf*HSP70‐X, *Hs*HSP70 and *Hs*HSC70 under steady‐state conditions. To allow easy comparison between each pairing, we normalised stimulation activity against the basal activity of each HSP70 (set to 100%, data on absolute fluorescent readings can be found in Data S4). J‐domain‐HSF1 fusions derived from all three JDP (PFA^JDS^, PFB^JDS^ and PFE^JDS^) were all able to significantly stimulate the ATPase activity of all three HSP70s (Fig. 2A–C). The highest level of stimulation was seen for the combination of *Hs*HSP70 with PFA^JDS^ (219% ± 25.2%, Fig. 2A). Both PFB^JDS^ and PFE^JDS^ could also stimulate *Hs*HSP70 to a high degree of significance (180% ± 14.3% and 176% ± 20.0%, respectively, Fig. 2B,C). Stimulation of *Hs*HSC70 was seen to be highest in combination with PFB^JDS^ (162% ± 10.6%) but was also noted for PFA^JDS^ and less significantly PFE^JDS^ (129% ± 6.4% and 115% ± 7.8%, respectively, Fig. 2A–C). Stimulation of *Pf*HSP70‐X was highest in combination with PFB^JDS^ (173% ± 15.6%), of a similar significance in combination with PFA^JDS^ (133% ± 15.7%) and lowest (but still significant) with PFE^JDS^ (120% ± 10.1%, Fig. 2A–C). When using J‐domain fusions containing the QPD mutation, which should ablate specific stimulation, observed ATP hydrolysis was either absent or significantly lower than that measured when using the wild‐type HPD partner (Fig. 2A–C). As a further control, we assayed the stimulatory effect on all HSP70s of adding only the HSF1 peptide. In all three cases, no significant increase in activity could be detected, verifying that the ATPase hydrolysis we observed in our assays was not due to HSP70 stimulation via HSF1 binding alone (Data S5A). Additionally, we assayed the ATPase activity of the J‐domain fusions without any HSP70 and observed no activity above background levels (data not shown).

### HSP70 ATPase stimulation by recombinant J‐domains under single turnover conditions

**Fig. 3:**
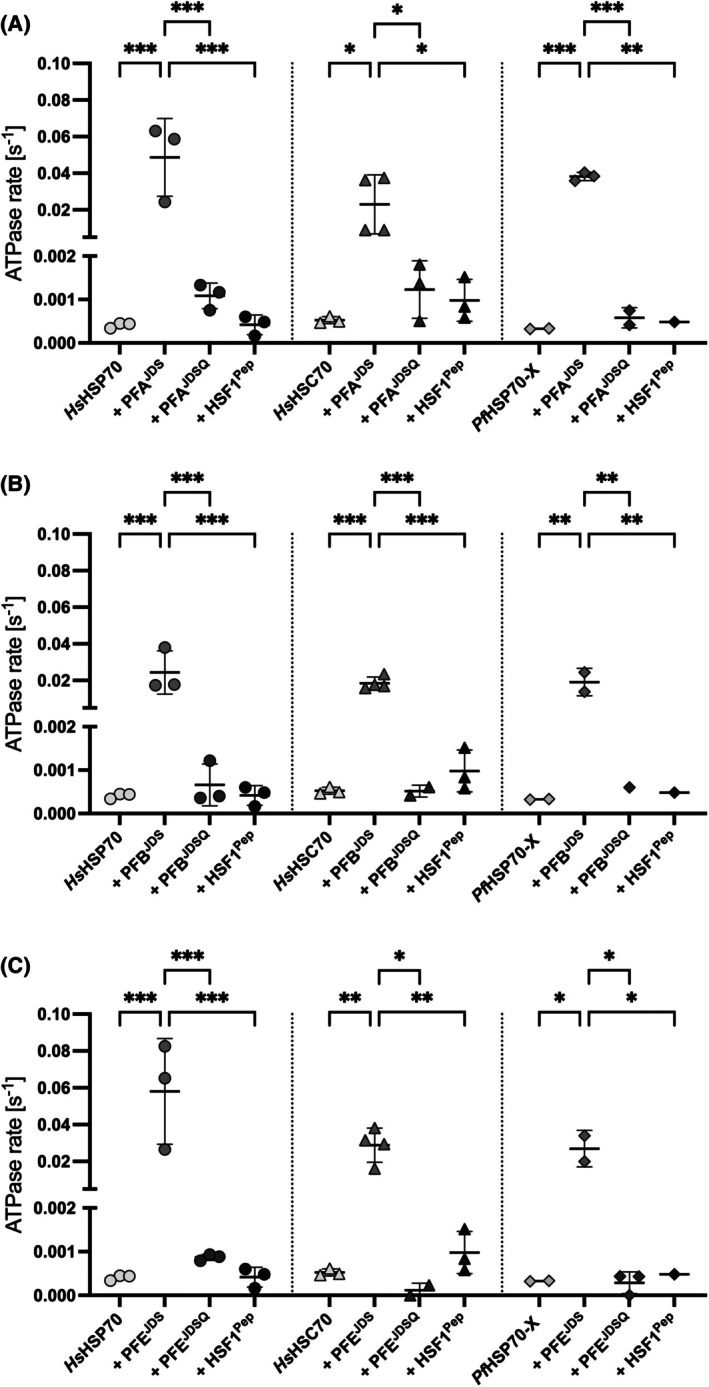
Stimulation of HSP70 ATPase activity by *Pf*JD (single turnover conditions). For all assays containing a functional J‐domain fusion *n* ≥ 2, with error bars indicating SD. Statistics were performed with ordinary one‐way ANOVA with Holm‐Šídák's multiple comparisons modification. *P* > 0.05 = nonsignificant (ns); *P* < 0.05 = *; *P* < 0.01 = **; *P* < 0.001 = ***. All assays included a control using a nonfunctional mutated version of the J‐domain containing a QPD motif (JDSQ), and a peptide only control (HFS1^pep^). The *Y*‐axis has been split to encompass the data range. (A) Stimulation of HSP70s by PFA^JDS^. (B) Stimulation of HSP70s by PFB^JDS^. (C) Stimulation of HSP70s by PFE^JDS^.

All previous data on stimulation of HSP70s by *Pf*JDP used steady‐state ATPase assays, but these are limited in their ability to precisely measure ATPase rates of individual protein pairings and can lead to an underestimation of stimulatory effects. In a physiological setting, the JDP/HSP70 cycle is additionally regulated by auxiliary factors such as nucleotide‐exchange factors (NEFs), which aid in resetting the HSP70 by removal of ADP following ATP hydrolysis. In the infected erythrocyte, it is not known whether NEFs are present and functional, and whether they can thus have an influence on the dynamics of the functional *Pf*JDP‐HSP70 interaction. For this reason, to allow us to determine ATPase rates independent of this rate‐limiting step, we carried out single‐turnover analysis. To our knowledge, this is the first time this technique has been applied to the study of chaperone/cochaperone dynamics in the malaria system. In all nine HSP70/J‐domain fusion pairings (3 J‐domains, 3 HSP70), a significant increase in the ATPase rate could be observed (Fig. 3A–C). The highest absolute stimulation could be observed for *Hs*HSP70 in combination with PFE^JDS^ (142 ± 70.1‐fold, Fig. 3C), but highly significant stimulation could also be observed when pairing this chaperone with PFA^JDS^ (119 ± 52.0‐fold) and PFB^JDS^ (60 ± 28.9‐fold, Fig. 3A,B). *Hs*HSC70 could be significantly stimulated by PFA^JDS^, PFB^JDS^ and PFE^JDS^ to comparable levels (44 ± 30.7, 35 ± 6.5 and 55 ± 17.7‐fold change, respectively, Fig. 3A–C). Likewise, *Pf*HSP70‐X could be stimulated by all three J‐domain fusions (117 ± 6.9, 58 ± 23.1 and 83 ± 30.6‐fold change, respectively, Fig. 3A–C). Stimulation of all HSP70s by the QPD mutants or HSF1 peptide alone was absent or at significantly lower levels from that observed with the wild‐type fusions (Fig. 3A–C), verifying that the measured effect is due only to a functional J‐domain interacting with HSP70.

## Discussion

Since the discovery of the *P. falciparum* exportome, many researchers have endeavoured to understand the function of these proteins. Many of these proteins have been shown to be involved in host cell modification, a process that is as advantageous to the parasite as it is dangerous to the host [3]. The majority of exported proteins have no homologues outside the genus *Plasmodium*, and thus no *in silico* predictions can be made on their specific molecular function [7]. This is not the case for exported *Pf*JDP, as the role of J‐domain proteins is well‐characterised in other systems, and usually involves a functional association with members of the HSP70 chaperone family. Generally, organisms encode more JDPs than HSP70s (e.g. 49 JDPs versus 7 *bona fide* HSP70s in human cells [32]), allowing a more diverse set of potential substrates to be recruited to HSP70. This is particularly significant in the case of exported *Pf*JDP, which have been suggested to act as ‘adapter proteins’ allowing the recognition of parasite‐encoded substrates by human HSP70s, a fascinating example of host–parasite interaction [16, 33]. The subfamily of exported Type II *Pf*JDP comprised of PFA66, PFE55 and PFB90 have high sequence and structural homology and are all expressed during similar stages in the parasite's life cycle, begging the question as to the reason behind this evolutionary expansion [15]. The substrate binding domain (SBD) of PFE55 determines its subcellular localisation at the J‐dots, raising the possibility that differential localisation of the three homologues may lead to functional diversity [34]. However, while PFB90 has indeed been localised to the knobs, all data to date suggest that PFA66 and PFE55 have an identical localisation at the J‐dots, negating this premise [16]. Additionally, a knockout of *pfa66* leads to dramatic changes in host cell modification, while a *pfe55* knockout appears phenotypically normal, suggesting an as yet unknown factor driving specificity [4, 19]. This led us to question whether the J‐domain itself may confer specificity of action, likely via the action of its partner HSP70(s). Several isolated *in vitro* attempts have been made to quantify these functional interactions using steady‐state ATPase assays; however, they were not able to distinguish between nonphysiological substrate/chaperone and true J‐domain/chaperone interactions [18, 19, 24, 25]. These publications also studied single JDP/HSP70 pairs in isolation, making it difficult to come to a solid conclusion about the relative stimulatory potential of *Pf*JDP on different HSP70s. This is especially relevant as the ATPase assays were performed in different laboratories using different recombinant proteins and different assay conditions. For this reason, we decided to carry out a systematic examination of the stimulation potential of three isolated J‐domains on three HSP70s, using an experimental design and controls, which can exclude non‐native interactions. Our additional use of single‐turnover assays removes the necessity for nucleotide exchange that is the rate‐limiting step in the reaction cycle, to gain further insight into reaction dynamics. Our data reveal that all three J‐domains have the potential to stimulate the ATPase activity of one exported, parasite‐encoded HSP70 (*Pf*HPS70‐X) and two residual human HSP70s (*Hs*HSP70, *Hs*HSC70) in the iRBC.

**Fig. 4:**
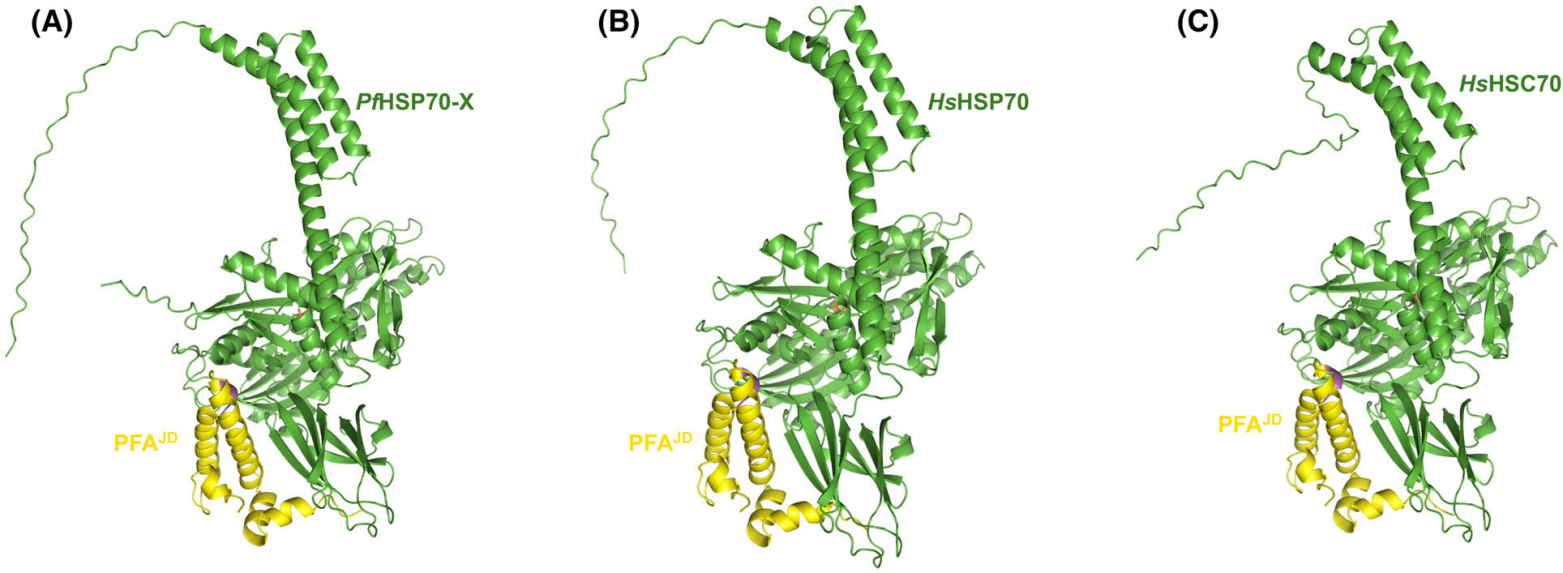
AlphaFold 3 modelling of three full‐length HSP70s with PFA^JD^. A: *Pf*HSP70‐X; B: *Hs*HSP70; C: *Hs*HSC70. The HPD motif is highlighted in light purple. ADP is shown in a stick representation. Modelling of all JD/HSP combinations can be found in Data S6.

There are subtle differences in the amount of stimulation observed for each *Pf*JD/HSP70 pairing, but the absolute level of stimulation is comparable to that previously determined for better characterised JD/HSP70 pairings known to be physiologically relevant [10, 29, 30, 35], that can reach two orders of magnitude or above in single turnover assays. This suggests that the *Pf*JDs here studied are extremely effective at stimulating all three studied HSP70, and that this stimulation is cell biologically relevant in the infected erythrocyte. Of the three HSP70 studied, *Hs*HSP70 appears to be most receptive to stimulation by the parasite‐derived J‐domains, while *Hs*HSC70 and *Pf*HSP70‐X respond at lower levels. Basal ATPase activity of *Hs*HSP70 was determined to be lower than that of *Hs*HSC70 and *PfHSP70‐X*; however, this is unlikely to be an explanation for the observed stimulation preference observed above, as, under our assay conditions, all three HSP70 populations are unlikely to be fully stimulated and thus have not yet met their maximal ATPase activity threshold, estimated (for DnaK, the *E. coli* homologue of HSP70) to be close to three orders of magnitude [10, 36]. AlphaFold 3 modelling of the interaction of isolated J‐domains with the three HSP70 proteins used in this study suggests no dramatic differences in association or positioning of the HPD motif relative to the HSP70 (example shown in Fig. 4, full modelling set in Data S6, statistics and further information in Data S7). Therefore, structural modelling alone does not reveal any obvious basis for the differential stimulation experimentally observed but does not exclude more local effects. Potentially, mutagenesis could be used to further experimentally interrogate this.

The J‐domains at the centre of this current study showed no obvious differences in their comparative stimulatory behaviour, although the statistical significance of their stimulatory effects differed. This suggests some specificity of individual J‐domains for a specific HSP70; however, we do wish to note that the inherent scattering of the data obtained from both ATPase assays does not allow strong conclusions to be drawn. Due to the phenotypic differences between a *pfa66* and *pfe55* knockout noted in previous sections, despite apparent identical localisation, we were particularly interested in any differential stimulatory effects between the J‐domains. A direct analysis of this data reveals no statistical differences between the behaviour of these two J‐domains (Data S8). This suggests that, despite similar localisations and J‐domains, the mechanistic specificity of these two proteins must be conferred by other domains.

One potentially, but unlikely, confounding factor could be the differential affinity of the HSF1‐peptide to the different HSP70s, in particular *Pf*HSP70‐X, as the affinity of the substrate to an HSP70 could influence the stimulatory effect [37]. The substrate specificity of *Pf*HSP70‐X has not been determined to our knowledge.

From a parasite cell biological perspective, the most interesting finding is that the *Pf*JD seem to have a higher stimulatory effect on *Hs*HSP70 compared to the other HSP70s studied. While a role for human HSP70s in host cell modification processes has been suggested for many years, no studies have defined which specific human HSP70 homologue might be most important, also due to difficulties in generating or obtaining reagents that are specific for individual homologues. There is proteomic evidence for the presence of several human HSP70s homologues in the infected erythrocyte [20], and *Hs*HSP70 has previously been found crosslinked with the *Pf*JDP PFE55 [23], but whether *Hs*HSP70 is the only residual human HSP70 playing an important role has remained unclear. Our data, while not excluding a role for (for example) *Hs*HSC70 in host cell modification, do suggest that as far as host cell modification is concerned, not all residual human HSP70s are equally important.

A limitation of our study is the lack of an endogenous JDP/HSP70 substrate. Binding of a substrate to HSP70 may have an influence on the subsequent stimulation by a JDP, and therefore we cannot exclude that the presence of a substrate in our assays could lead to different results. So far, no endogenous substrate for either *Pf*JDPs or HSP70s in the erythrocyte has been identified. Given the difference in phenotype between a *PFA66* and *PFE55* knockout, we suggest that a final appraisal of the relative importance of substrate binding and differential ATPase stimulatory effects on specific human HSP70 is likely to rely on complementation experiments in cell culture, swapping the substrate binding and J‐domains between these two closely related proteins. Alternately, identification of potential substrates for exported *Pf*JDP via BioID may also help to clarify the situation.

## Conflict of interest

The authors declare no conflict of interest.

## Author contributions

JMP was involved in conceptualisation and project administration. JMP and JB were involved in formal analysis. JB, MK, L‐HR, JE, DP were involved in investigation. BMK, MPM, JMP were involved in methodology. MPM and JMP were involved in supervision. JB and JMP were involved in writing—original draft. JB, MK, MPM, JMP were involved in writing—review and editing.

## Supporting information


**Data S1.** Amino acid sequence alignment of Type II J‐domain proteins.
**Data S2**. AlphaFold 3 prediction of the structure of mature PFA66, PFB90 and PFE55.
**Data S3**. Representative images of SDS/PAGE gels of all chaperones and fusion proteins.
**Data S4**. Absolute stimulation of the HSP70 ATPase activity by *Plasmodium falciparum* JD‐HSF1^pep^ fusion proteins (steady state conditions).
**Data S5**. Steady state controls.
**Data S6**. Modelling of full‐length HSP70s with J‐domains.
**Data S7**. Statistics for AlphaFold3 modelling.
**Data S8**. Estimation plots and t‐test to compare stimulative capabilities of PFA^JDS^ vs. PFE^JDS^ (single turnover condition).
**Data S9**. List of primers and sequences used in this study.

## Data Availability

The data supporting the findings of this study are available within the article and its supplementary material.
