# Supplementary material for: Promiscuous stimulation of HSP70 ATPase activity by parasite‐derived J‐domains
Source: FEBS Open Bio. 2026 Jan 31;16(7):1293–304. doi: 10.1002/2211-5463.70207 (PMC13327048; doi:10.1002/2211-5463.70207)
Supplement: Supplementary file 1 — Data S1. Amino acid sequence alignment of Type II J‐domain proteins. Data S2. AlphaFold 3 prediction of the structure of mature PFA66, PFB90 and PFE55. Data S3. Representative images of SDS/PAGE gels of all chaperones and fusion proteins. Data S4. Absolute stimulation of the HSP70 ATPase activity by Plasmodium falciparum JD‐HSF1pep fusion proteins (steady state conditions). Data S5. Steady state controls. Data S6. Modelling of full‐length HSP70s with J‐domains. Data S7. Statistics for AlphaFold3 modelling. Data S8. Estimation plots and t‐test to compare stimulative capabilities of PFAJDS vs. PFEJDS (single turnover condition). Data S9. List of primers and sequences used in this study. [file FEB4-16-1293-s001.pdf]

```
CLUSTAL O(1.2.4) multiple sequence alignment


NP_006136.1         ------------------MGKDYYQTLGLARGASDEEIKRAYRRQALRYHPDKNKEP--- 39
PF3D7_0113700       AEGNKNFFFNKDNGVFGKSSMDYYTLLGVDKGCSEDDLRRAYLKLAMKWHPDKHVNKGSK 60
PF3D7_0201800       AQTQRNFKSKNG-KASTKKNEDYYSILGVSRDCTNEDIKKAYKKLAMKWHPDKHLNAASK 59
PF3D7_0501100       ----AEFNSGSSRESSKTDETDYYAVLGLTKDCTQDDIKKAYRKLAMKWHPDKHLNDEDK 56
                                         ***  **: :..:::::::** : *:::****: :

NP_006136.1         -GAEEKFKEIAEAYDVLSDPRKREIFDRYGEEGLKGSGPSGGSGGGANGTSFSYTFHGDP 98
PF3D7_0113700       VEAEEKFKNICEAYSVLSDNEKRVKYDLFGMDALKQSGFNSSNFQG--------NISINP 112
PF3D7_0201800       KEADNMFKSISEAYEVLSDEEKRDIYDKYGEEGLDKYGSNNGHSKG--------FKRTDP 111
PF3D7_0501100       VEAERKFKLIGEAYEVLSDEEKRKNYDLFGQSGLGGTTTNDEAYYT--------YSNIDP 108
                      *:. ** * ***.**** .**  :* :* ..*     ..                 :*

NP_006136.1         HAMFAEFFGGRNPFDTFFGQRNGEEGMDIDDPFSGFPMGMGGFTNVNFGRSRSAQE---- 154
PF3D7_0113700       LEVFTKAYSFYNKY---FSKSSGAGNHNIF----------------THIKNLYPLRNDFS 153
PF3D7_0201800       NDVFSKFFKTETKF---YSNSPSSPNGNVLFEGSL----FGGSSPFSGINPRSGSGYTTS 164
PF3D7_0501100       NELFSRFFSHDASS---FFSQGF-------DDFPS----FQGFASMNSRRPRSSRSNIFS 154
                      :*:. :         : .                          .  .

NP_006136.1         --PARKKQDPPVTHDLRVSLEEIYSGCTKKMKISHKRLNPDGKSIRNEDKILTIEVKKGW 212
PF3D7_0113700       EDESSYNDVEEYEVPLYVTLEDLYNGCTKTLKVTRKRY--DGCYLYYEDYFINVDIKQGW 211
PF3D7_0201800       KSFSSMDKVEEYVVPLYVTLEDLYNGTQKKLKVTRKRC--QGVTTYDDEFFVTVDIKSGW 222
PF3D7_0501100       R---SFGRAASFEVPLQVTLEELYTGCRKKLKVTRKRF--VGLNSYEDNTFITVDVKPGW 209
                                   * *:**::*.*  *.:*:::**    *     :: ::.:::* **

NP_006136.1         KEGTKITFPKEGDQTSN-NIPADIVFVLKDKPHNIFKRDGSDVIYPARISLREALCGCTV 271
PF3D7_0113700       NNGTKITFHGEGDQSSPDSYPGDLVLVLQTKKHSKFVRKSRDLYYRHIITLEQSLTGFDF 271
PF3D7_0201800       CDGTTITYKGEGDQTSPMSNPGDLVFTIKTVDHDRFVRSYNDLIYRCPITLEQALTGHKF 282
PF3D7_0501100       SEGTKINFHGEGEQSSPNEQPGDLVFIIKTKPHDRFIREGNNLIYKCYLPLDKALTGFQF 269
                     :**.*.:  **:*:*  . *.*:*: ::   *. * *.  :: *   : * ::* *  .

NP_006136.1         NVPTLDGRTIPVVFKDVIRPGMRRKVPGEGLPLPKTPEKRGDLIIEFEVIFPERIPQTSR 331
PF3D7_0113700       VIKSLDNRDIHIQIDEVVKPDTKKVIKNEGMPYSRDPSIRGNLIVEFDIIYPNTIKKEQK 331
PF3D7_0201800       TIITLDNRDIDIQVDEIVTPLTTRVITSEGMPYMENPKMKGNLIIEFDIIFPKKLSDEQK 342
PF3D7_0501100       SIKSLDNRDINVRVDDIINPNSKKIITNEGMPYSKSPSVKGDLFIEFDIVFPKKLSPEQK 329
                     : :**.* * : ..::: *   : : .**:*  . *. :*:*::**:::*: :    .:

NP_006136.1         TVLEQVLPI--  340
PF3D7_0113700       KLIKEIFKESY  342
PF3D7_0201800       ELIKEALGGNG  353
PF3D7_0501100       RTLKETLENTY  340
                      ::: :
```

Supporting Information 1. Amino acid sequence alignment of Type II J-domain proteins. Full-length amino acid sequence alignment of the Type II J-domain proteins PFA66 (PF3D7_0113700), PFB90 (PF3D7_0201800), PFE55 (PF3D7_0501100) and the human homolog DnaJB1 (NP_006136.1) performed with CLUSTAL 0(1.2.4). For the exported *Plasmodium falciparum* J-domain proteins, the N-terminal signal peptide and PEXEL motif were removed.

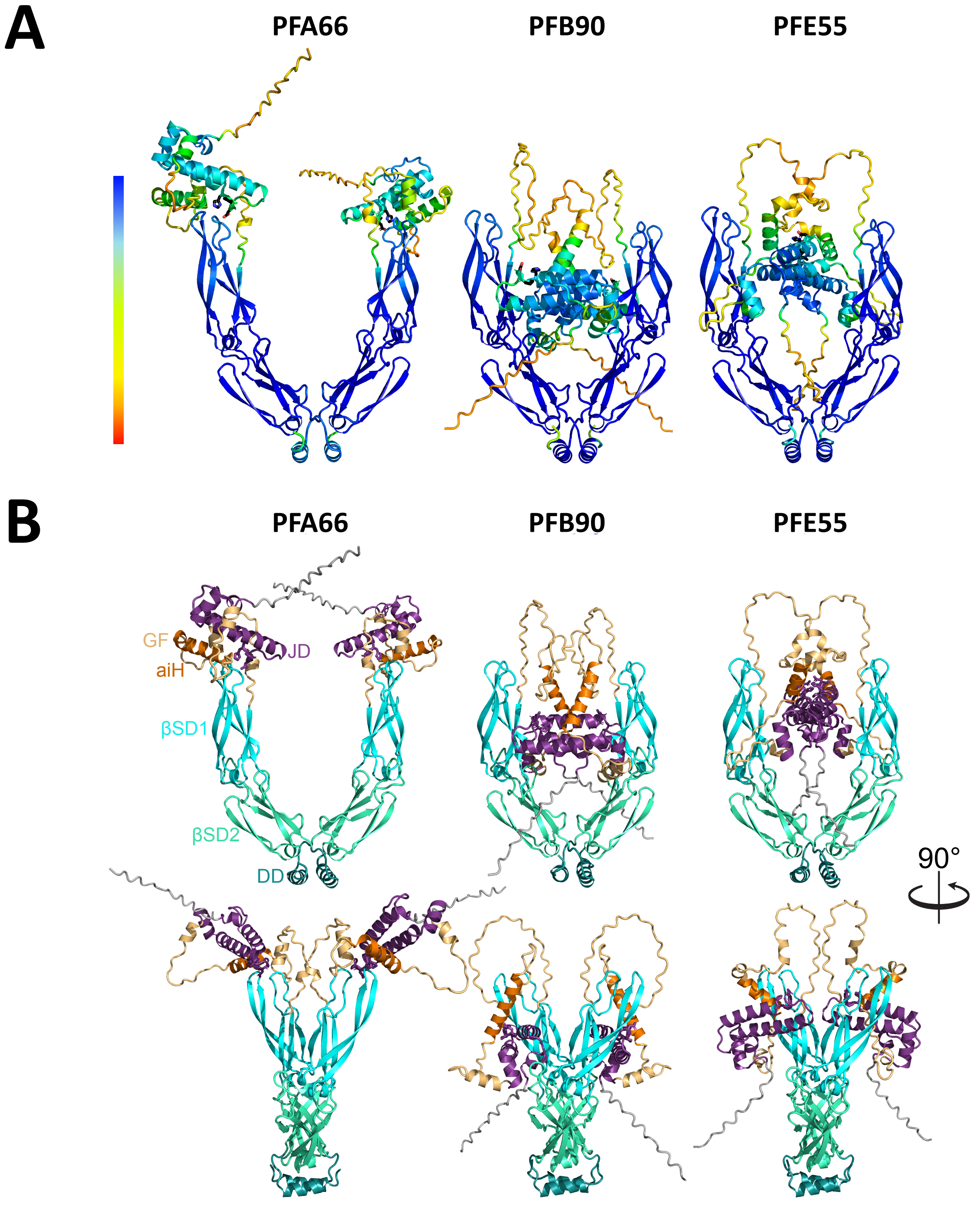

Supporting Information 2: AlphaFold 3 prediction of the structure of the mature PFA66, PFB90 and PFE55. *(A)* Secondary structure representation of the predicted structures of PFA66, PFB90 and PFE55 colored according to the prediction confidence with red low confidence and blue high confidence as indicated in the color scale. *(B)* Secondary structure representation of the predicted structures of PFA66, PFB90 and PFE55 colored according to domains with J-domain (JD) in violet purple, glycine-phenylalanine-rich region (GF) in light orange, autoinhibitory helix (aiH) in orange, β-sandwich domain (βSD) 1 in cyan, βSD2 in green cyan, and dimerisation domain (DD) in deep teal. The position of the JD is likely arbitrary as they are connected to the well structured βSDs by a long, largely intrinsically disordered, highly flexible GF.

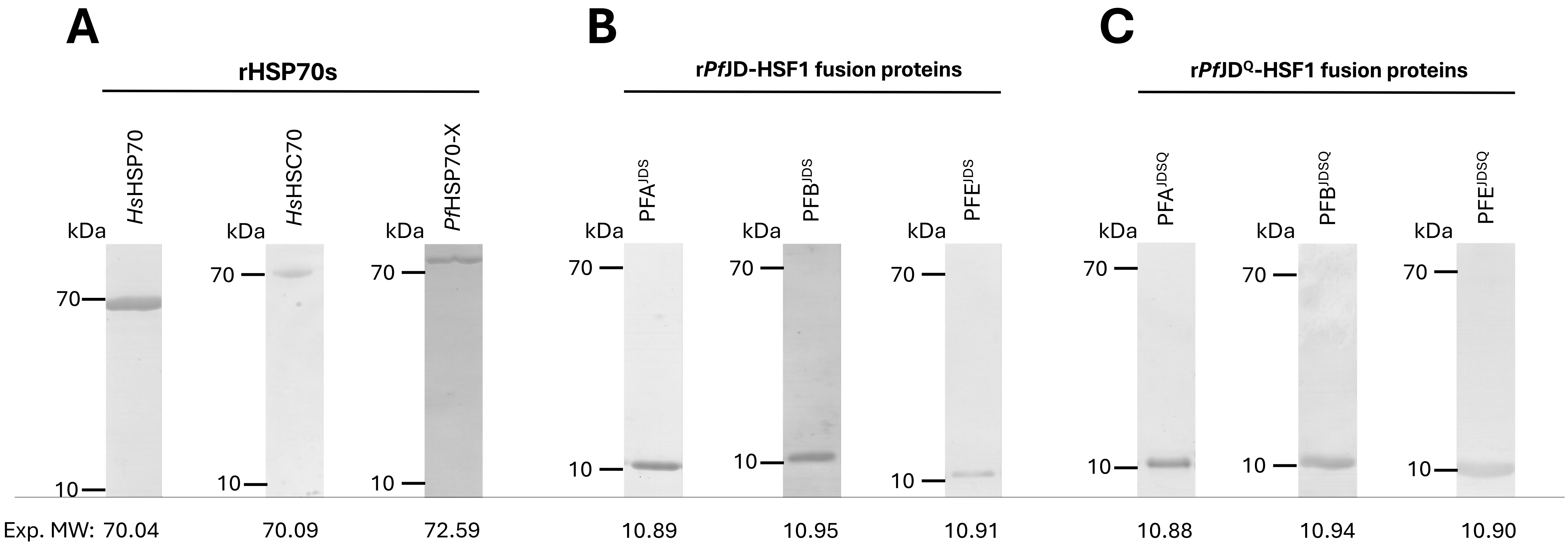

Supporting Information 3. Representative images of SDS-PAGE gels of all chaperones and fusion proteins. *(A)* Recombinant full-length *Hs*HSP70, *Hs*HSC70 and *Pf*HSP70-X. *(B)* Recombinant *Plasmodium falciparum* J-domains of PFA66, PFB90 and PFE55 fused to HSF1 peptide (S461-Q471). *(C)* Recombinant *Plasmodium falciparum* J-domains of PFA66, PFB90 and PFE55 with HPD → QPD mutation fused to HSF1 peptide (S461-Q471). The respective expected molecular weight (kDa) is noted.

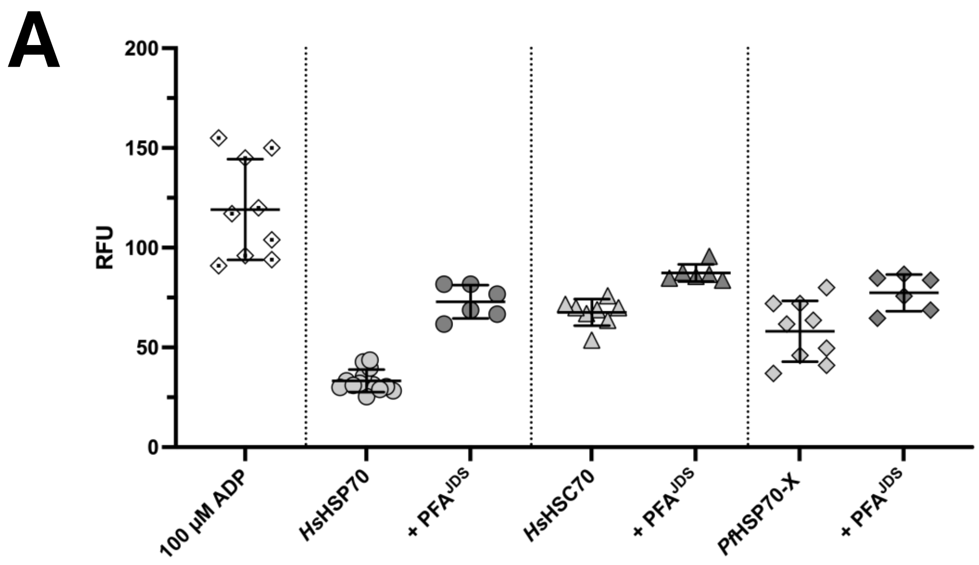

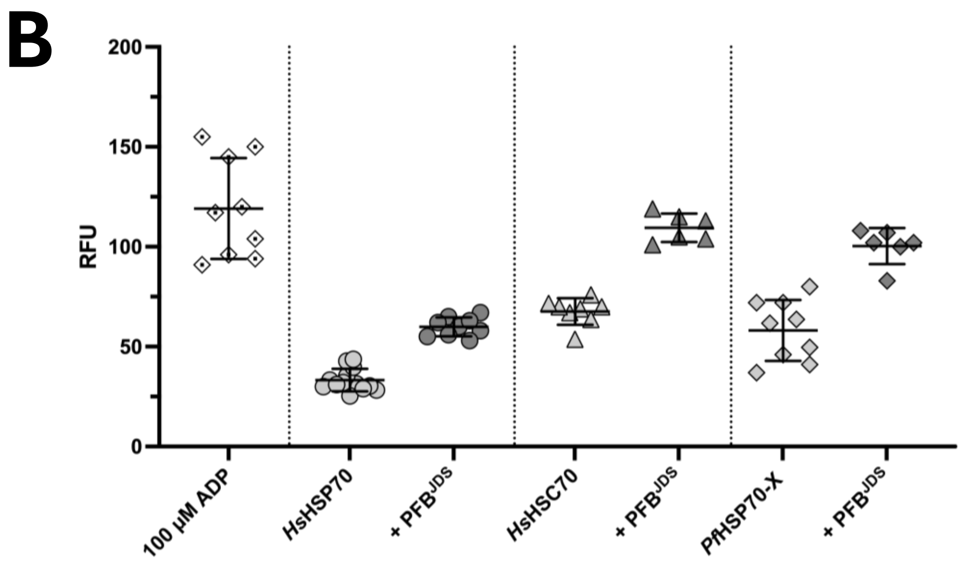

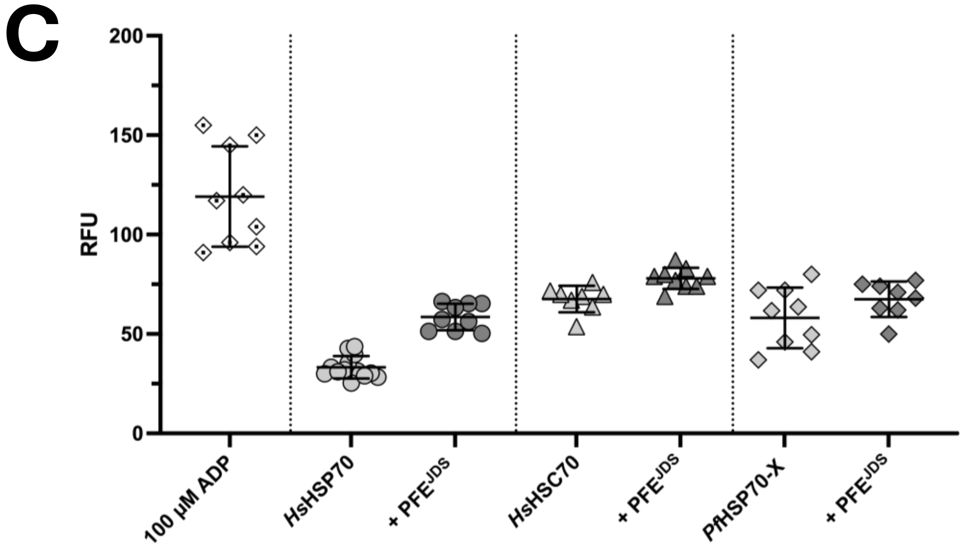

Supporting Information 4. Absolute stimulation of the HSP70 ATPase activity by *Plasmodium falciparum* JD-HSF1^{pep} fusion proteins (steady state conditions). Provided is the read-out data of fluorescence intensity measurements as relative fluorescence units (RFU). For all experiments n ≥ 6, with error bars indicating SD. As reference, the 100 µM ADP signal simulates the expected RFU strength for complete hydrolysis of 100 µM ATP as used in this assay. The basal measurement of *Hs*HSP70 gives the weakest RFU signal compared the both *Hs*HSC70 and *Pf*HSP70-X (A-C). However, PFA^{JDS} and PFE^{JDS} can stimulate *Hs*HSP70 to comparable absolute ATP hydrolysis levels as respectively stimulated *Hs*HSC70 and *Pf*HSP70-X (A, C), whereas PFB^{JDS} stimulates *Hs*HSC70 and *Pf*HSP70-X to higher absolute ATP hydrolysis (B).

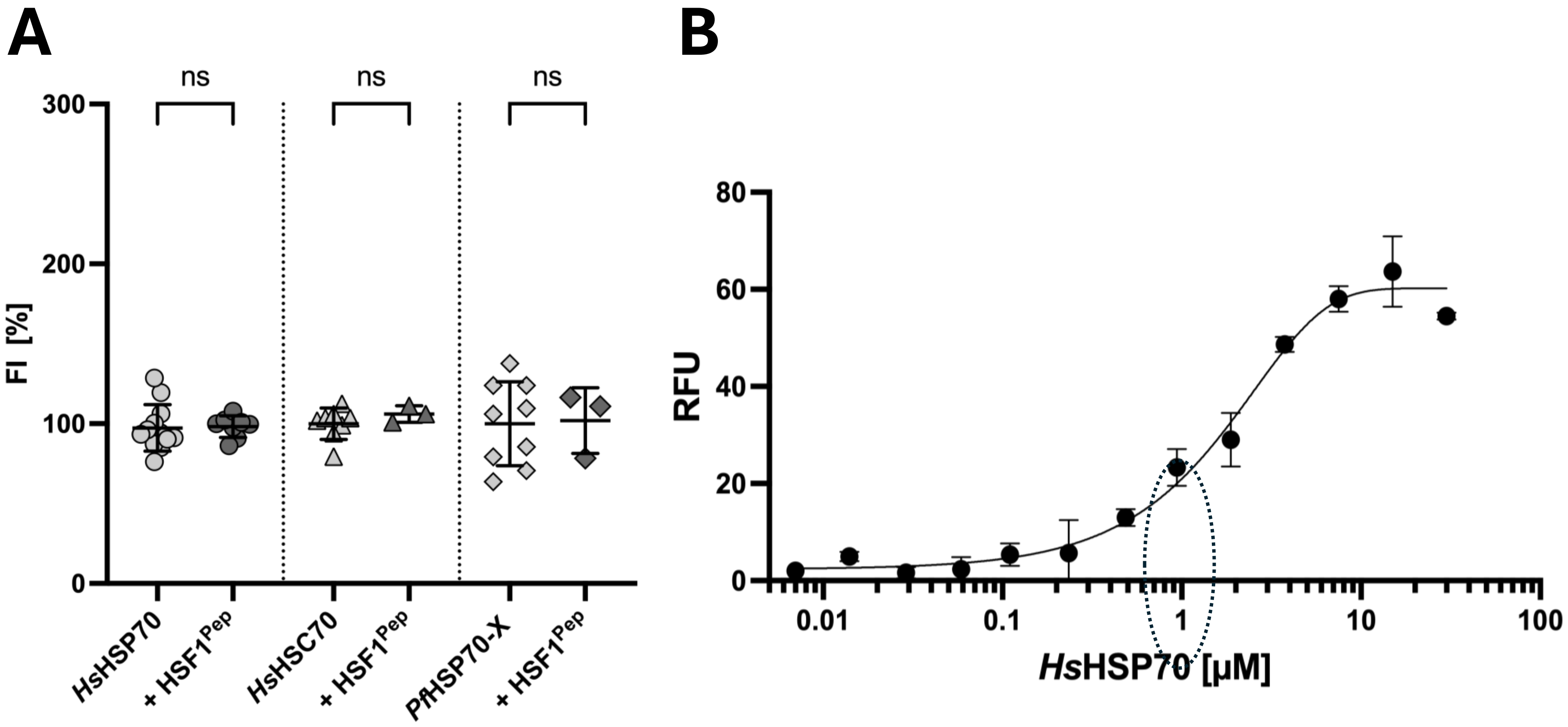

Supporting Information 5. ***(A)*** **ATPase activity stimulation of HSP70s by HSF1 peptide (steady state conditions).** All values were normalized against the basal ATP hydrolysis activity of the respective HSP70 (set to 100 %). This negative control was performed to test whether the HSF1^{Pep} (S461-Q471) has a stimulatory effect on the ATPase activity of the HSP70s. For neither *Hs*HSP70, *Hs*HSC70 nor *Pf*HSP70-X was a significant relative increase of fluorescence intensity and thus hydrolyzed total ATP (due to the presence of HSF1^{pep}) observed. Statistics were performed with one-way ANOVA with Holm-Šídák's multiple comparisons modification. For HSF1^{pep} n = 3 (*Hs*HSC70 and *Pf*HSP70-X controls) or n = 9 (*Hs*HSP70). For HSP70s, n = 8 (*Hs*HSC70), n = 9 (*Pf*HSP70-X) or n = 12 (*Hs*HSP70). Error bars indicate SD. $p > 0.05$ = non-significant (ns); $p < 0.05$ = *; $p < 0.01$ = **; $p < 0.001$ = ***. ***(B)*** **Basal *Hs*HSP70 titration curve (steady state condition).** To define a suitable HSP70 concentration, a titration curve ranging from 2,7 x 10^{-5} µM to 2,3 x 10^{2} µM *Hs*HSP70 was performed. Depicted is data from fluorescence intensity measurements in RFU, with n = 3. Error bars indicate SD. In order to detect stimulation of ATP hydrolysis by JD-HSF1^{pep} fusion proteins, the subsequently used steady-state ATPase assay was conducted with HSP70 at a concentration of 1 µM.

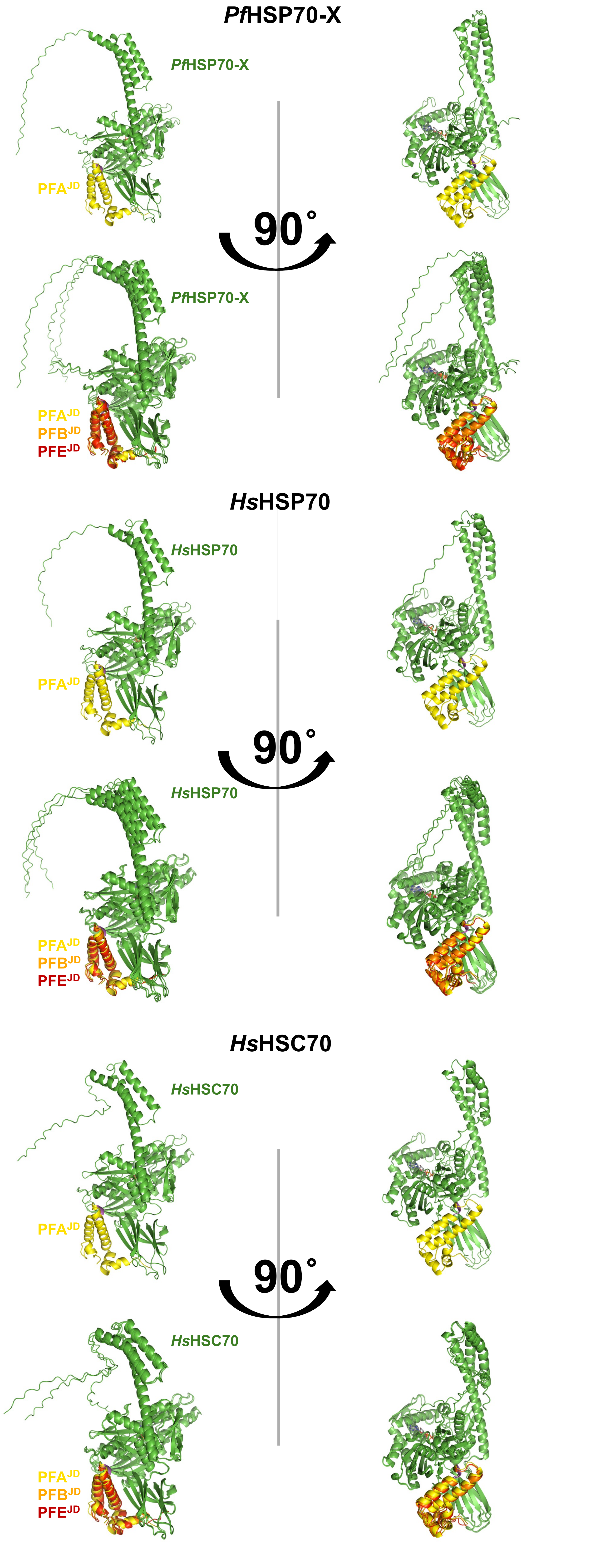

Supporting Information 6. Modelling of full-length HSP70s with J-domains. In each panel, the upper images shows a model of PFA^{JD} (chosen as the crystal structure is available), the lower including shows an overlay of three separate models including each JD (colour code as labels). The HPD motif is highlighted in light purple. ADP is shown in a stick representation.

Supporting information 7
Modelling Statistics

Promiscuous stimulation of HSP70 ATPase activity by parasite-derived J-domains.

Julian Barth[1], Moritz Koch[2], Le-Han Rössner[3], Johanna Eichhorn[1], Denys Pogoryelov[1,4], Matthias P Mayer[2], Jude M Przyborski[1*]

[1]Biochemistry and Molecular Biology, Justus Liebig University, Giessen, Germany. [2] Center for Molecular Biology (ZMBH), Heidelberg, Germany. [3]Institute of Botany, Justus Liebig University, Giessen, Germany. [4]Institute of Pharmaceutical Chemistry, Goethe-University Frankfurt, Frankfurt am Main, Germany.

***For correspondence: Jude. M. Przyborski** jude.przyborski@ernaehrung.uni-giessen.de

Table S1: AlphaFold3 Quality Metrics for HSP/C70–J-domain Complex Models

Model (Complex)	pTM[1]	ipTM[2]	Ranking score[3]	Disordered fraction[4]	Recycles[5]	Clashes[6]
*Pf*HSP70_jda	0.78	0.86	0.88	0.08	10	0
*Pf*HSP70_jdb	0.78	0.87	0.88	0.06	10	0
*Pf*HSP70_jde	0.78	0.86	0.88	0.08	10	0
*Hs*HSC70_jda	0.80	0.87	0.88	0.06	10	0
*Hs*HSC70_jdb	0.81	0.88	0.89	0.04	10	0
*Hs*HSC70_jde	0.80	0.86	0.88	0.06	10	0
*Hs*HSP70_jda	0.80	0.86	0.87	0.06	10	0
*Hs*HSP70_jdb	0.81	0.87	0.88	0.04	10	0
*Hs*HSP70_jde	0.80	0.86	0.87	0.06	10	0

Explanatory notes

[1] pTM: predicted TM-score, confidence of overall structural fold. [2] ipTM: interface predicted TM-score, confidence of inter-chain interactions. [3] Ranking score: AlphaFold3 combined confidence metric (weighted pTM + ipTM). [4] Disordered fraction: predicted proportion of intrinsically disordered residues. Region-specific analysis revealed that the HSP/C70 core domains were predicted with very high per-residue confidence (expected pLDDT > 90), supporting their structural stability. By contrast, the flanking C-termini of the HSP/C70 and J-domains (Table S1) showed lower predicted confidence (pLDDT < 70), in line with the higher fraction of disordered residues. High confidence core (HSP/C70 and J-domain, pLDDT >90) and flexible/disordered termini HSP/C70-J-domain, (~4 – 8%), *Pf*HSP70 (residues 1-11 and 624-658), *Hs*HSC70 (residues 615-646), *Hs*HSP70 (residues 614-641), PFA^{JD} (residues 75-83), PFB^{JD} (residues 78-83), PFE^{JD} (residues 74-83). [5] Recycles: number of refinement iterations used in AlphaFold3 prediction. [6] Clashes: predicted steric clashes (0 = none detected).

Structural confidence of AlphaFold3-predicted HSP70–J-domain complexes

The AlphaFold3 predictions for nine HSP/C70–J-domain complexes yielded consistently high global confidence scores (pTM values of 0.78–0.81 and ipTM values of 0.86–0.88; Table S1). Ranking scores ranged between 0.87 and 0.89, confirming the robustness of the modeled complexes. The fraction of residues predicted as disordered was low (4 – 8%), and no steric clashes were detected.

Together, these results demonstrate that AlphaFold3 provides reliable structural models for HSP/C70–J-domain complexes, while also highlighting the C-terminal flexibility in the J-domains as a potential determinant of functional differences.

Quality Assessment

Taken together, the AlphaFold 3 metrics confirm that the modeled HSP70–J-domain complexes are structurally reliable and suitable for graphical representation and interpretation. The observed differences between domain variants are thus likely meaningful and not artifacts of prediction instability.

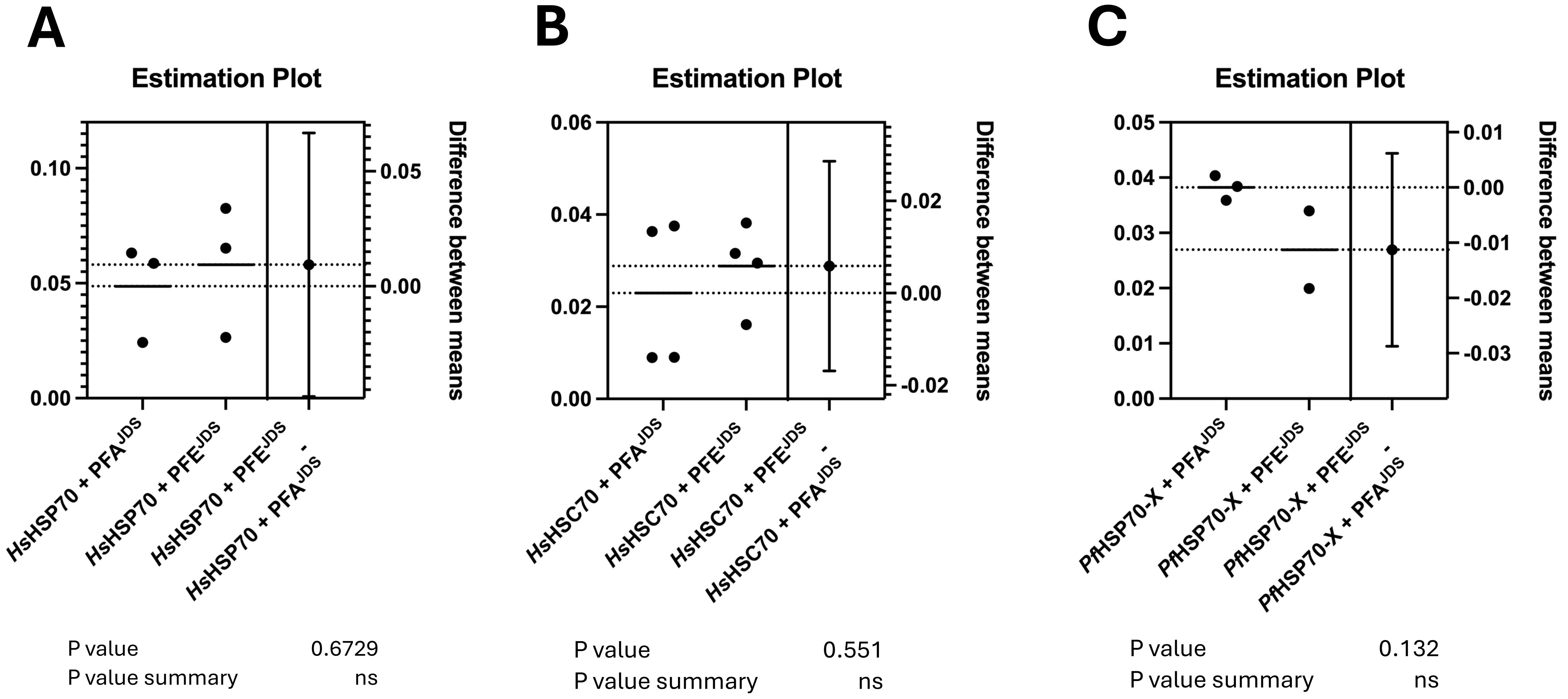

Supporting Information Figure 8. Estimation plots and t-test to compare stimulative capabilities of PFA^{JDS} vs. PFE^{JDS} (single turnover condition). To assess whether the stimulation of ATPase rate of *Hs*HSP70 (A), *Hs*HSC70 (B), and *Pf*HSP70-X (C) by either PFA^{JDS} or PFE^{JDS} differs significantly under single-turnover conditions, an unpaired two-tailed t-test was performed. In the estimation plots, the left panels display individual measurements together with group means (A–C), while the right panels depict the mean differences along with their 95% confidence intervals (A–C). Statistical significance was defined as $p > 0.05$ = non-significant (ns); $p < 0.05$ = *.

Supporting Information 9. List of primers and sequences used in this study.

Name (internal)	Sequence
PFAJ_MSCI_F #1	5’ ATAATGGCCAGCATGGATTACTATACCCTGCTGGG 3‘
PFA2_JDS_SewR1 #2	5‘ GCTGTTTGCCACTACCGCCGCTGTTAAAGCCGCTCTGCTTCAGCGC 3‘
PFAJ_Fus_SalI_R2 #3	5’ CTACGTCGACTTACTGCGCCGTGTACTGAACCAGCTGTTTGCCACTACCGCC 3‘
PFA_JDS_MSCI_F #1	5‘ TTATATTGGCCAATGGATTATTATACCCTGCTGGGC
PFA_JDS_SEW_R #2	5‘ CACATGTTTATCCGGCTGCCATTTCATCGC 3‘
PFA_JDS_SEW_F #3	5‘ GCGATGAAATGGCAGCCGGATAAACATGTG 3‘
PFA_JDS_SALI_R #4	5‘ TAAGTCGACTTTTTCAAACTGCGGATGGCTCC 3‘
PFA2JDS_QPD_r #2	5’ GGCGATGAAATGGCAGCCGGATAAACACGTG 3‘
PFA2JDS_QPD_f #3	5‘ CACGTGTTTATCCGGCTGCCATTTCATCGCC 3‘
PFBJ_MscI_F #1	5‘ AATTATGGCCATGGATTATTATTCCATATTAGGTGTTAGTAG 3‘
PFBJ_R1 #2	5’ CTGTTTGCCACTACCGCCATTATTAGAACCATATTTATCTAATCCTTC 3‘
PFB_E_SalI_R2 #3	5‘ TTAAGTCGACTTACTGCGCCGTGTACTGAACCAGCTGTTTGCCACTACCGCC 3‘
PFB_QPD_SEW_F #2	5’ GCTATGAAGTGGCAGCCTGATAAACACTTAAATGC 3‘
PFB_QPD_SEW_R #3	5’ GCATTTAAGTGTTTATCAGGCTGCCACTTCATAGC 3‘
PFE_MscI_F #1	5’ AATTATGGCCATGGATTACTATGCTGTATTAGGTCTTAC 3‘
PFEJ_R1 #2	5’ CTGTTTGCCACTACCGCCATCGTTAGTTGTAGTTCCTCCTAATCC 3‘
PFE_QPD_SEW_F #3	5‘ GCATATAGAAAACTGGCTATGAAATGGCAGCCTGATAAGCACCTAAATGACGAAG 3‘
pSUMO_forward	5‘ GGACTCCTTAAGATTCTTGTACG 3‘
pSUMO_rev_seq	5‘ CTTTCGGGCTTTGTTAGCAGCC 3‘
